# Dr. Emmelin Pickette Philbrick

**Published:** 1916-12

**Authors:** 


**Dr. Emmelin Pickette Philbrick** (not listed in State Directory nor Polk), died Nov. 9, at the home of George T. Luce of Warsaw, aged 78. She had practiced medicine in Warsaw and Pike for several years.

